# Supplementary material for: Suitability of Gelatin Methacrylate and Hydroxyapatite Hydrogels for 3D-Bioprinted Bone Tissue
Source: Materials (Basel). 2024 Mar 6;17(5):1218. doi: 10.3390/ma17051218 (PMC10934138; doi:10.3390/ma17051218)
Supplement: Supplementary file 1 [file materials-17-01218-s001.zip › materials-2814559-supplementary.pdf]

Supplementary Materials

Suitability of Gelatin Methacrylate and Hydroxyapatite Hydrogels for 3D-Bioprinted Bone Tissue

Paul Stolarov, Jonathan de Vries, Sean Stapleton, Lauren Morris, Kari Martyniak and Thomas J. Kean *

Biionix Cluster, Department of Internal Medicine, College of Medicine, University of Central Florida, Orlando, FL 32827, USA
* Correspondence: thomas.kean@ucf.edu

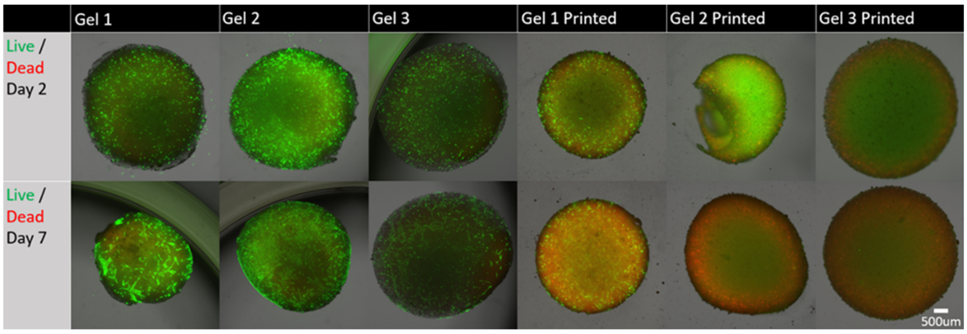

Figure S1. Cell viability in GelMA/HA bioinks: Live hMSCs stained green and dead hMSCs stained red; composite images are shown. Gels 2 and 3 printed were not quantifiable and were not analyzed for cell viability.
